# Fatty Acids Predominantly Affect Anti-Hydroxyl Radical Activity and FRAP Value: The Case Study of Two Edible Mushrooms

**DOI:** 10.3390/antiox8100480

**Published:** 2019-10-12

**Authors:** Maja Karaman, Kristina Atlagić, Aleksandra Novaković, Filip Šibul, Miroslav Živić, Katarina Stevanović, Boris Pejin

**Affiliations:** 1 Department of Biology and Ecology – DBE, Faculty of Sciences – PMF, University of Novi Sad, Trg Dositeja Obradovića 2, 21000 Novi Sad, Serbia; maja.karaman@gmail.com; 2 Institute of Physiology and Biochemistry, Faculty of Biology, University of Belgrade, Studentski trg 16, 11158 Belgrade, Serbia; mzivic@bio.bg.ac.rs (M.Ž.); katarina.stevanovic@bio.bg.ac.rs (K.S.); 3 Institute for Food Technology – FINS, Bulevar cara Lazara 1, 21000 Novi Sad, Serbia; aleksandra.novakovic72@gmail.com; 4 Department of Chemistry, Biochemistry and Environmental Protection, Faculty of Sciences – PMF, University of Novi Sad, Trg Dositeja Obradovića 3, 21000 Novi Sad, Serbia; filip.sibul@dh.uns.ac.rs; 5 Department of Life Sciences, Institute for Multidisciplinary Research – IMSI, University of Belgrade, Kneza Višeslava 1, 11030 Belgrade, Serbia

**Keywords:** *Coprinus comatus*, *Coprinellus truncorum*, submerged cultivation, phenolic and fatty acid profiles, antioxidant potential

## Abstract

Compared to plants, nowadays mushrooms attract more attention as functional foods, due to a number of advantages in manipulating them. This study aimed to screen the chemical composition (fatty acids and phenolics) and antioxidant potential (OH•, 2,2-diphenyl-1-picrylhydrazyl (DPPH•) and ferric reducing ability of plasma (FRAP)) of two edible mushrooms, *Coprinus comatus* and *Coprinellus truncorum,* collected from nature and submerged cultivation. Partial least square regression analysis has pointed out the importance of some fatty acids—more precisely, unsaturated fatty acids (UFAs) followed by fatty acids possessing both short (C6:0 and C8:0) and long (C23:0 and C24:0) saturated chains—and phenolic compounds (such as protocatechuic acid, daidzein, *p*-hydroxybenzoic acid, genistein and vanillic acid) for promising anti-OH•, FRAP and anti-DPPH• activities, respectively. However, other fatty acids (C16:0, C18:0 and C18:3n3) along with the flavonol isorhamnetin are actually suspected to negatively affect (by acting pro-oxidative) the aforementioned parameters, respectively. Taken together, design of new food supplements targeting oxidative stress might be predominantly based on the various UFAs combinations (C18:2n6, C20:1, C20:2, C20:4n6, C22:2, C22:1n9, etc.), particularly if OH• is suspected to play an important role.

## 1. Introduction

Antioxidant activity (AO) is considered to be an important feature of edible mushrooms in the prevention of oxidative stress [1,2,3]. Their antioxidants may well contribute to maintaining their physiological balance by neutralisation of free radicals (with stress on reactive oxygen species), without toxic or mutagenic effects, as opposed to synthetic antioxidants [3,4]. Generally speaking, it is believed that phenolics are primarily responsible for AO [1,5,6]. These compounds may originate either from fruiting bodies (generative structures) or from mycelia and extracellular broth (i.e., from the filtrate of a submerged culture, vegetative structures) [7]. Submerged cultivation of macrofungi represents a biotechnological process for their growth under controlled conditions aiming to provide valuable biomass and extracellular metabolites [8]. The medium composition, pH, temperature and aeration are of crucial importance for expressing fungal biopotential in submerged cultivation [9]. Similarly, biopotential of the shoots from nature is affected by habitat and growth stage [1,10,11]. The edible mushrooms *Coprinus comatus* and *Coprinellus truncorum* are widely distributed in Asia, Europe and America. They are both considered as well-balanced nutraceuticals [7,12,13,14,15,16,17]. Thus, we aimed to screen the AO of their methanolic extracts under in vitro conditions, versus chemical composition (fatty acids and phenolics), using both shoots from nature (i.e., fruiting body (FB)) and submerged cultivation (cultured mycelium (M) and fermentation broth (filtrate F)).

## 2. Materials and Methods

### 2.1. Biological Material

Wild growing *Coprinus comatus* (O.F. Müll.) Pers. 1797 (Ph. Basidiomycota, Cl. Agaricomycetes, O. Agaricales, Fam. Agaricaceae) was sampled at meadow near Sremski Karlovci (town in Northern Serbia), while *Coprinellus truncorum* (Scop.) Redhead, Vilgalys & Moncalvo 2001 (Ph. Basidiomycota, Cl. Agaricomycetes, O. Agaricales, Fam. Psathyrellaceae) was collected at grassland in Novi Sad (town in Northern Serbia). The relevant voucher specimens (12-00704 and 12-00705 for *C. comatus* and *C. truncorum*, respectively) were deposited at the Herbarium of the Department of Biology and Ecology – DBE, Faculty of Sciences – PMF, University of Novi Sad. Finally, mycelia isolated from the FB of both species and cultivated at 26 °C, for 10 days, on Malt Agar (Torlak, Serbia), were deposited in the culture collection of the ProFungi Laboratory (Department of Biology and Ecology – DBE, Faculty of Sciences – PMF, University of Novi Sad).

### 2.2. Submerged Cultivation and Preparation of Extracts

After cultivation on Malt Agar, 5 plugs of isolated mycelia were transferred into 100 mL of fermentation broth that contained 5 g peptone, 35 g glucose, 5 g yeast extract, 1 g K_2_HPO_4_, 0.5 g MgSO_4_·7H_2_O and 0.05 g vitamin B, at pH 6.51. After incubation on a rotary shaker at 100 rpm, 26 °C, for 14 days (IKA KS 4000i control, Werke GmbH & Co.KG, Staufen, Germany), the biomass was filtrated (Fioroni Filter, AHLSTROM Group, Ingré, France). Afterwards, both the mycelia biomass and filtrate were lyophilised to dryness (CHRIST ALPHA 2-4 LDplus, Freeze Dryer, Martin Christ Gefriertrocknungsanalgen GmbH, Osterode am Harz, Germany). All extracts from the both species (FB, M and F) were prepared as previously described [1].

### 2.3. Determination of Antioxidant Activity

AO of the examined extracts was assessed by following in vitro spectrophotometric assays: anti-2,2-diphenyl-1-picrylhydrazyl (anti-DPPH) radical activity (DPPH assay), anti-hydroxyl (anti-OH) radical activity (OH assay) and ferric reducing ability of plasma (FRAP assay). All assays were performed in triplicate, while the results were expressed as the mean values ± standard deviations.

### 2.4. DPPH Assay

Anti-DPPH radical activity of the extracts was determined as previously described [18]. 2,2-Diphenyl-1-picrylhydrazyl (DPPH) was from Fluka Chemie (Fluka Chemie GmBH, Buchs, Switzerland). The reaction mixture contained 60 μL of 90 μM DPPH, 180 μL methanol and 10 μL of the relevant mushroom extract. After incubation (for 30 min in the dark, at room temperature), the absorption was measured at 515 nm (Multiscan, Thermo Scientific, Waltham, MA, USA). The results were expressed as IC_50_ values (μg/mL) obtained from the calculated RSC (Radical Scavenging Capacity) values, by following formula:

RSC _(DPPH)_ (%) = (1 − A_sample_/A_control_) × 100% (1)

where A_sample_ and A_control_ stand for the absorbance of the tested and control samples, respectively. A lower IC_50_ value corresponds to higher AO of the sample.

### 2.5. OH Assay

Anti-OH radical activity was determined according to a modified method of Halliwell and Gutteridge [19]. The reaction mixture contained 100 μL H_2_O_2_, 100 μL FeSO_4_, 100 μL 2-deoxyribose-D-ribose, 2.7 mL of phosphate buffer pH 7.4 and 10 μL of the each extract. After incubation (60 min at 37 °C), 0.2 mL of EDTA (ethylenediaminetetraacetic Acid) and 2 mL of TBA reagent (5.2 mL perchloric acid, 1.5 g thiobarbituric acid and 60 g of trichloroacetic acid) were added. Afterwards, the absorbance of a characteristic pink complex was measured at 532 nm. Finally, the results were expressed as IC_50_ values ± standard deviations (μg/mL).

### 2.6. FRAP Assay

Ferric reducing ability of plasma (FRAP) was evaluated by a spectrophotometric assay as previously described [20]. The reaction mixture contained 225 μL of FRAP reagent (10 mmol/L TPTZ solution in 40 mmol/L HCl, 0.02 mmol/L FeCl_3_·6H_2_O and acetate buffer (pH 3.6), in a ratio 10:1:1), 22.5 μL of distilled water and 10 μL of each extract. After 6 min of incubation, the absorbance was measured, while reduction potential was calculated as milligrams of ascorbic acid equivalents (AAE) per gram of dry weight (mg AAE/g d.w.), calculated according to the standard calibration curve of ascorbic acid solution.

### 2.7. Determination of Phenolic Compounds by HPLC-MS/MS Analysis

This chemical analysis was done applying the method of Orčić et al. [21]. All extracts were diluted with mobile phase solvents A (water) and B (methanol), premixed in 1:1 ratio, to obtain a final concentration of 2 mg/mL. Reference standards of the phenolic compounds were obtained from Sigma-Aldrich Chem (Steinheim, Germany), Fluka Chemie gmbh (Buchs, Switzerland) or from ChromaDex (ChromaDex Corp., Santa Ana, CA, USA). HPLC gradient grade methanol was purchased from J. T. Baker (Deventer, The Netherlands), and p.a. formic acid and DMSO from Merck (Darmstadt, Germany). A total of 15 working standards, ranging from 1.53 ng/mL to 2.50 × 10^4^ ng/mL, were prepared by serial 1:1 dilutions of the standard mixture with solvents A and B (1:1). Samples and standards were analysed using Agilent Technologies 1200 Series high-performance liquid chromatograph coupled with Agilent Technologies 6410A Triple Quad tandem mass spectrometer with electrospray ion source (Agilent Technologies, Inc., Santa Clara, CA, USA), and controlled by Agilent Technologies MassHunter Workstation software – Data Acquisition (ver. B.03.01, Agilent Technologies, Inc., Santa Clara, CA, USA). First, 5 µL were injected into the system. Afterwards, compounds were separated on Zorbax Eclipse XDB-C18 (50 mm × 4.6 mm, 1.8 µm, Agilent Technologies, Inc., Santa Clara, CA, USA) rapid resolution column held at 50 °C. Mobile phase was delivered at flow rate of 1 mL/min in gradient mode (0 min 30% B, 6 min 70% B, 9 min 100% B, 12 min 100% B, re-equilibration time 3 min). Eluted compounds were detected by ESI-MS, using the ion source parameters as follows: nebulisation gas (N_2_) pressure 40 psi, drying gas (N_2_) flow 9 L/min and temperature 350 °C, capillary voltage 4 kV, negative polarity. Data were acquired in dynamic MRM mode, using the optimised compound-specific parameters (retention time, precursor ion, product ion, fragmentor voltage, collision voltage) as reported by Orčić et al. [21]. For all the compounds, peak areas were determined using Agilent MassHunter Workstation software – Qualitative Analysis (ver. B.04.00, Agilent Technologies, Inc., Santa Clara, CA, USA). Briefly, calibration curves were plotted in the OriginLabs Origin Pro (ver. 8.0) software (Northampton, MA, USA). Limit of detection (LoD) was estimated as the lowest concentration resulting in well-defined peak [21].

### 2.8. GC-MS Identification and Quantification of Fatty Acids

This chemical analysis was also performed as previously described [22]. As a solvent, *n*-heptane was used, along with the evaporation in the nitrogen stream. The prepared samples were analysed on a GC Agilent 7890A system (Agilent Technologies, Santa Clara, CA, USA) equipped with a Flame Ionisation Detector (FID) and an auto-injecting liquid system on a capillary column of mixed silica (Supelco SP-2560 Capillary GC Column, 100 m × 0.25 mm, d = 0.20 μm, Merck KGaA, Darmstadt, Germany). The gas carrier was helium of purity of 99.9997%, at a flow rate of 1.5 mL/min and a pressure of 1.092 bar. The samples were injected in a column in split mode in the ratio 30:1. The applied temperatures ranged from 40 to 230 °C. Total time of analysis was 41.311 min. Fatty acid methyl ester peaks were identified by comparing retention times (RI) from RI samples of the Supelco 37 component fatty acid methyl ester mix standard as well as by the internal data obtained in the pre-assay of fatty acids in a GC with a mass detector. The obtained results were expressed as the mass of the individual fatty acid or group of fatty acids (g) in 100 g of fatty acids from the biological material.

### 2.9. Statistical Analysis

All measurements were performed in triplicate. The results were expressed as the mean values ± standard deviations. IC_50_ values were obtained by interpolation from a linear regression analysis using OriginLabs Origin Pro (ver. 8.0) software. One-way analysis of variance (ANOVA) with Tukey’s test was used to determine the statistically significant difference between the analysed extracts (*p* < 0.01). The strength of association between pairs of variables was measured with the Pearson product moment correlation at a 5% level of significance (*p* < 0.05). Partial Least Squares Regression (PLSR) was applied for multivariate analysis (XLSTAT statistical and data analysis solution, Addinsoft 2019, Boston, MA, USA).

## 3. Results and Discussion

### 3.1. Anti-DPPH Radical Activity

The submerged mushroom extracts of both species were more potent compared to FB extracts (Table 1). In comparison, *C. comatus* FB ethanolic extract exhibited much lower anti-DPPH radical activity (IC_50_ 2.56 ± 0.31 mg/mL) [13]. The similar case is with the polysaccharide extract of *C. comatus* FB [23]. Furthermore, *C. comatus* FB methanolic extract originating from Lipovica Forest near Belgrade (Serbia) did show a neglected activity (IC_50_ 3.76 ± 0.48 mg/mL) [14], compared to the one reported herein (IC_50_ 172.74 ± 7.10 µg/mL) for *C. comatus* FB methanolic extract. Also, two submerged cultivated *C. comatus* mycelial isolates, namely *C. comatus* 906 and *C. comatus* 1021, exhibited by far lower activities (EC_50_ values 1.1 ± 0.2 and 2.2 ± 0.3 mg/mL, and 2.6 ± 0.4 and 3.5 ± 0.4 mg/mL for water and ethanolic extracts, respectively) [23], though both strains were cultivated practically under the same conditions as reported herein.

### 3.2. Anti-OH Radical Activity

*C. truncorum* FB extract, the sample displaying the most potent anti-OH radical activity, was followed by *C. comatus* FB extract (Table 1). In comparison, *C. comatus* FB extract (collected in China) had much lower activity (3.23 ± 0.28 mg/mL) [5].

### 3.3. FRAP Value

Once again FB extracts were more effective. The most profound FRAP value was recorded for *C. comatus* FB extract. A number of other mushroom species including *Xylaria polymorpha* (3.25 ± 0.04 mg AAE/g d.w.), *Meripilus giganteus* (10.45 ± 0.44 mg AAE/g d.w.) and *Agrocybe aegerita* (10.74 ± 0.09 mg AAE/g d.w.) exhibited lower FRAP values [1]. Generally speaking, differences in the antioxidant potential of different samples were clearly observed; some of the tested samples were actually proven to be more effective compared to the methanolic extracts of some previously analysed species [1,2,5,14,23].

### 3.4. HPLC-MS/MS Determination of Phenolic Compounds

Following HPLC-MS/MS procedure optimised for the quantification of 45 phenolics, 28 compounds were identified in the tested samples (Table 2). *p*-Hydroxybenzoic acid was the most abundant compound, followed by quinic acid. Both phenolics are known as good antioxidants, due to their reducing properties (depending on hydrogen or electron donors) and ability to stabilise the unpaired electron [24,25]. In addition to this, protocatechuic acid was also found in all the samples, with notably greater amount in the submerged extracts, with stress on *C. comatus* F extract. Both in vitro and in vivo designed studies have clearly pointed out that protocatechuic acid may be considered as effective antioxidant, even more potent than trolox, a synthetic vitamin E analog [26,27,28]. Furthermore, cinnamic acid was detected in the most of the extracts (except *C. truncorum* M extract). On the other hand, the isoflavonoids daidzein and genistein, also proven antioxidants [29,30,31], were detected only in the submerged cultures. Finally, vanillic acid, another antioxidant of natural origin [32,33], was identified only in the submerged extracts.

### 3.5. GC-MS Analysis

Gas Chromatography–Mass Spectrometry (GC-MS) was used to analyse the contents of fatty acids. A total of 28 fatty acids were identified in the screened extracts (Table 3). The content of total unsaturated fatty acids (UFAs), mono-unsaturated fatty acids (MUFAs), poly-unsaturated fatty acids (PUFAs) and saturated fatty acids (SFAs) revealed that UFAs were most abundant ones. The same fatty acids are recommended as high-quality ingredients of a healthy diet, inter alia, capable of decreasing blood lipids [13,16,17]. In the majority of the analysed samples (except *C. truncorum* F extract), linoleic acid (C18:2n6c) was the most common one. Additionally, oleic fatty acid (C18:1n9c) was present in all the samples.

These findings are in a good agreement with literature data [13,14,15,34,35]. However, no one has previously reported *C. truncorum* FB fatty acid profile, to the best of our knowledge. The aforementioned profile is somewhat similar to *C. micaceus* fatty acid profile [36].

Also, these are real pioneering data for the both mushrooms samples developed in the submerged cultivation. Furthermore, it’s noteworthy to mention that FB extracts contained more UFAs and PUFAs, compared to the rest of samples (Table 3). Thus far, UFA content has been linked with AO increase [37]. On the other hand, PUFAs have been claimed to modulate the activity of antioxidant enzymes. However, their AO cannot be easily predicted, since it doesn’t depend on the length of the carbon chain and/or degree of unsaturation [17].

### 3.6. Partial Least Squares Regression (PLSR) Analysis

**Figure 1:**
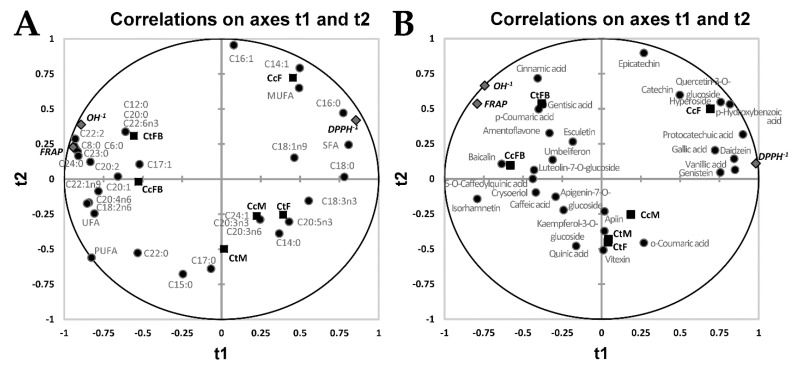
Partial least squares regression (PLSR) analysis for antioxidant potential. (**A**) Correlations between the AO activity of all extracts and their fatty acid profiles; (**B**) Correlations between the AO activity of all extracts and their phenolic profiles; (t_1_, t_2_)—first two PLSR components.

Partial least squares regression (PLSR) analysis was used to define the possible interrelationships between the chemical composition (based on their phenolic (Table 2) and fatty acid (Table 3) profiles) (independent variables, X) and AO activity (DPPH•, OH• and FRAP, dependent variables, Y; reciprocal values of IC_50_ for anti-DPPH and anti-OH radical activities, Table 1) of the analysed methanolic extracts. Firstly, PLSR was performed for all three AO measures, resulting in the correlation circles between the extracts, their fatty acid (Figure 1A) or phenolic (Figure 1B) profile and AO measures, with first two PLSR components (t_1_, t_2_). Although the global *R*^2^ between Y and (t_1_, t_2_) (which gives an upper bound of how well the model explains the data and predicts new observations) is slightly higher for the phenolic profile (0.962), compared to the fatty acid one (0.934), the quality of the former regression is lower since *R*^2^ resulting from the cross-validation (*Q*^2^cum), that defines the stability of the model and sets the lower bound of how well the model explains the data [38], is 0.389, compared to 0.686 for the fatty acid profile. Figure 1 shows that all dependent variables are located at the periphery of the correlation circle meaning that can be explained by the concentrations of fatty acid or phenolic compounds located either in their vicinity (e.g., C22:2 or cinnamic acid, in the case of OH•) exhibiting a positive (antioxidative) influence, or opposite to them (e.g., C18:3n3 or vanillic acid, in the case of OH•) displaying a negative (pro-oxidative) one.

**Figure 2:**
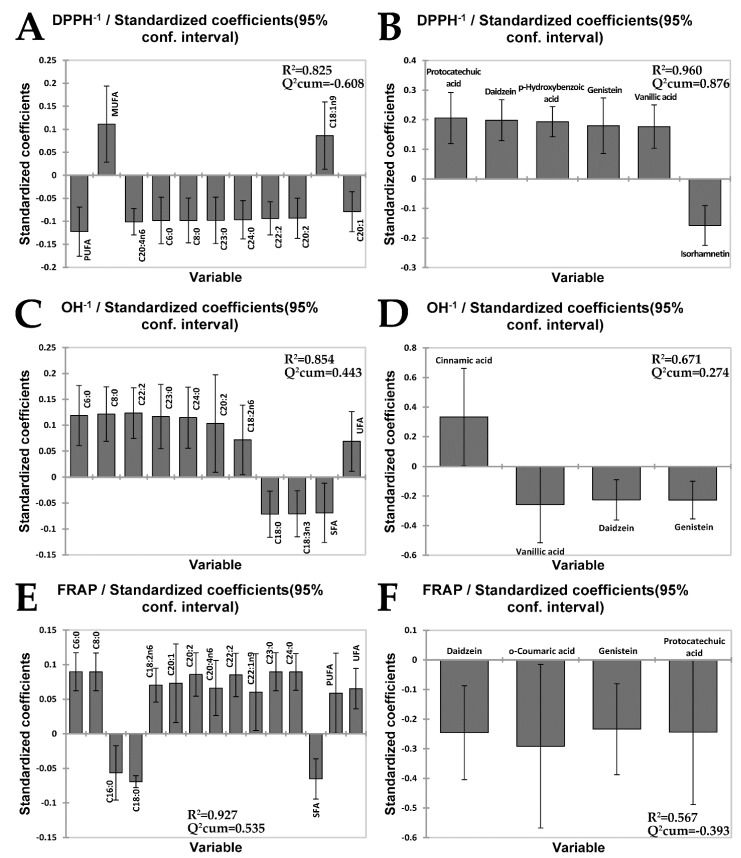
A separate one-component PLSR model for each dependent variable. The variation in the AO activity of the extracts in regard to fatty acid and phenolic profiles estimated by DPPH (**A**,**B**), OH (**C**,**D**) and FRAP (**E**,**F**) assays.

Although the correlation circle is useful for gaining the overall picture, it actually doesn’t indicate (specify) which combination of fatty acid or phenolic compounds has statistically significant influence on the specific measure of AO activity of the analysed mushrooms extracts. In order to estimate this, a separate one-component PLSR model was built for each dependent variable. The models were then pruned until all variables with insignificant standardised regression coefficients (confidence intervals include 0) were deleted. Bar graphs of the regression coefficients for all three models are shown in Figure 2. Upwards and downwards pointing bars indicate positive (antioxidative) and negative (pro-oxidative) influences, respectively. Comparison of *R*^2^ and *Q*^2^cum values clearly pointed out that AO activity of the extracts measured by FRAP assay was much better explained by PLSR model based on their fatty acid profiles (Figure 2E, *R*^2^ = 0.927, *Q*^2^cum = 0.535) versus the phenolic ones (Figure 2F, *R*^2^ = 0.567, *Q*^2^cum = −0.393). Practically, entire variability (92.7%) in the AO activity of the extracts measured by FRAP assay can be explained by coordinated antioxidative potential of unsaturated fatty acids (UFAs) (C18:2n6, C20:1, C20:2, C20:4n6, C22:2 and C22:1n9) followed by short (C6:0 and C8:0) or long (C23:0 and C24:0) chain saturated fatty acids (SFAs). Since palmitic and stearic acids were by far most abundant SFAs, SFAs generally may be linked with pro-oxidative action, unlike UFAs and PUFAs (Figure 2E). Similarly to FRAP, anti-OH radical activity of the extracts is also much better explained by PLSR model based on their fatty acid profiles (Figure 2C, *R*^2^ = 0.854, *Q*^2^cum = 0.443), compared to the phenolic ones (Figure 2D, *R*^2^ = 0.671, *Q*^2^cum = 0.274). Such a trend is actually expected due to a strong correlation between FRAP and OH• values (*R* = 0.932, *p* = 0.007), that is confirmed by their close position at the correlation circle (Figure 1A), too. In fact, due to such a tight correlation, OH• PLSR model is essentially based on the identical fatty acids as FRAP PLSR model (Figure 2C). Contrary to FRAP and OH assays, the PLSR model based on the phenolic profiles of the extracts (Figure 2B, *R*^2^ = 0.960, *Q*^2^cum = 0.876) much better explains their anti-DPPH radical activity, compared to the model based on the fatty acid profiles (Figure 2A, *R*^2^ = 0.825, *Q*^2^cum = −0.608). Protocatechuic acid, daidzein, *p*-hydroxybenzoic acid, genistein and vanillic acid are phenolics suspected to primarily contribute to anti-DPPH radical activity, unlike the flavonol isorhamnetin that is, indeed, likely to display pro-oxidative activity (Figure 2B).

## 4. Conclusions

Taken together, submerged *C. comatus* F extract was most effective in neutralising DPPH radicals, while *C. truncorum* & *C. comatus* FB extracts were most effective in neutralising OH radicals. The aforementioned FB extracts also displayed potent FRAP values. According to PLSR analysis, fatty acid chemistry is suspected to predominantly affect anti-OH radical activity and FRAP value, while phenolic chemistry is likely to be the key one for the observed anti-DPPH radical activity. Consequently, design of new food supplements targeting OH radicals might be predominantly based on the various UFAs combinations (C18:2n6, C20:1, C20:2, C20:4n6, C22:2, C22:1n9, etc.)

## Figures and Tables

**Table 1:** Antioxidant activity of the methanolic extracts of the mushrooms *Coprinus comatus* and *Coprinellus truncorum*. FB—Fruiting Body; M—Mycelium; F—Filtrate; DPPH—DPPH assay; OH—OH assay; FRAP—FRAP assay; AAE—ascorbic acid equivalents.

Extracts	DPPH (IC_50_) (μg/mL)	OH (IC_50_) (μg/mL)	FRAP (mg AAE g d.w.)
*C. comatus*			
FB	172.74 ± 7.10 ^f^	**6.65 ± 1.99 ^a^**	**81.05 ± 5.91 ^a^**
M	33.99 ± 5.48 ^b^	55.80 ± 12.13 ^d^	27.28 ± 2.58 ^c^
F	**22.34 ± 4.32 ^а^**	24.50 ± 13.70 ^b^	27.17 ± 2.63 ^c^
*C. truncorum*			
FB	96.66 ± 5.79 ^e^	**5.62 ± 1.21 ^a^**	**68.26 ± 2.71 ^b^**
M	54.98 ± 1.79 ^d^	69.06 ± 19.11 ^e^	29.92 ± 2.50 ^c^
F	**46.25 ± 4.38 ^c^**	41.90 ± 2.30 ^c^	14.01 ± 2.03 ^d^

^a,b,c,d,e,f^ Significant differences between extracts were determined by Tukey HSD test at *p* < 0.01. In each column different letters mean significant differences (Tukey, HSD, ANOVA). The difference is related both to the analysed species and samples (FB, M and F extracts). Bold values stand for the most promising biological activities.

**Table 2:** The content of phenolic compounds in the analysed samples (µg/g).

Class	Compound	Extracts					
		CcFB MeOH	CcM MeOH	CcF MeOH	CtFB MeOH	CtM MeOH	CtF MeOH
Flavones	Crysoeriol	0.168	0.131	n.d.	n.d.	n.d.	n.d.
	Vitexin	0.195	0.572	n.d.	n.d.	n.d.	0.300
	Apigenin-7-*O*-glucoside	0.437	0.424	0.183	0.120	0.112	0.255
	Luteolin-7-*O*-glucoside	0.362	0.162	0.168	0.103	0.103	0.168
	Apiin	n.d.	0.340	n.d.	0.170	n.d.	0.170
	Baicalin	3.96	n.d.	n.d.	n.d.	n.d.	n.d.
Flavonols	Isorhamnetin	3.90	2.84	2.25	2.69	2.69	2.77
	Kaempferol-3-*O*-glucoside	0.437	0.454	0.229	n.d.	0.140	0.281
	Hyperoside	n.d.	n.d.	0.106	n.d.	n.d.	n.d.
	Quercetin-3-*O*-glucoside	n.d.	n.d.	0.128	n.d.	n.d.	n.d.
Flavanols	Catechin	n.d.	6.79	12.7	13.1	4.71	6.61
	Epicatechin	n.d.	n.d.	8.70	8.70	n.d.	n.d.
Biflavonoids	Amentoflavone	0.294	n.d.	0.118	n.d.	n.d.	n.d.
Isoflavonoids	Daidzein	n.d.	**38.7**	**43.1**	n.d.	**2.94**	**9.01**
	Genistein	n.d.	**22.6**	**18.1**	n.d.	**1.64**	**3.48**
Hydroxybenzoic acids	*p*-Hydroxybenzoic acid	11.2	297	**752**	145	5.20	88.4
	Protocatechuic acid	1.71	25.0	**65.3**	3.76	4.87	20.6
	Vanillic acid	n.d.	**23.4**	**42.2**	n.d.	**29.1**	n.d.
	Gallic acid	n.d.	n.d.	7.93	n.d.	5.46	n.d.
	Gentisic acid	n.d.	0.264	n.d.	1.09	n.d.	n.d.
Hydroxycinnamic acids	Cinnamic acid	**29.7**	28.2	24.3	**66.8**	n.d.	19.4
	*p*-Coumaric acid	2.36	3.44	2.92	**40.6**	1.28	11.3
	*o*-Coumaric acid	n.d.	n.d.	0.384	n.d.	0.262	1.44
	Caffeic acid	0.664	0.664	0.664	1.88	1.81	1.39
Coumarins	Esculetin	n.d.	0.481	n.d.	0.511	n.d.	n.d.
	Umbelliferone	n.d.	n.d.	n.d.	1.04	0.936	n.d.
Cyclohexanecarboxylic acids	Quinic acid	**132**	37.3	5.77	104	**580**	87.7
Chlorogenic acids	5-*O*-Caffeoylquinic acid	2.17	0.641	0.481	n.d.	n.d.	0.641
Total		189.55	489.38	**987.73**	389.56	641.25	253.91

^a^ not detected—peak not observed, the content is lower than the LOD; CcFB—*C. comatus* fruiting body; CcM—*C. comatus* mycelia; CcF—*C. comatus* filtrate; CtFB—*C. truncorum* fruiting body; CtM—*C. truncorum* mycelia; CtF—*C. truncorum* filtrate; MeOH—methanolic extract. Bold values highlight the importance of the relevant contents.

**Table 3:** The content of fatty acid compounds in the analysed samples (relative %).

Fatty Acid Carbon Numbers	Common Names (Acid)	*C. comatus FB*	*C. truncorum FB*	*C. comatus M*	*C. comatus F*	*C. truncorum M*	*C. truncorum F*
		%					
C6:0	Caproic	0.21	0.16	n.d.	n.d.	n.d.	n.d.
C8:0	Caprylic	0.09	0.08	n.d.	n.d.	n.d.	n.d.
C12:0	Lauric	n.d.	0.05	n.d.	n.d.	n.d.	n.d.
C14:0	Myristic	0.40	0.17	0.24	n.d.	0.42	3.04
C14:1	Myristoleic	n.d.	n.d.	n.d.	12.52	n.d.	n.d.
C15:0	Pentadecanoic	0.31	0.17	0.50	n.d.	0.59	n.d.
C16:0	Palmitic	**13.32**	**12.39**	**13.54**	**28.58**	**16.15**	**22.85**
C16:1	Palmitoleic	0.83	2.04	0.52	3.74	0.36	n.d.
C17:0	Heptadecanoic	0.11	0.09	0.36	n.d.	0.30	n.d.
C17:1	Heptadecanoic (*cis*-10)	0.11	0.46	0.35	n.d.	n.d.	n.d.
C18:0	Stearic	0.78	0.92	2.73	**6.70**	2.66	**11.55**
C18:1n9c	Oleic	**4.32**	**9.08**	**20.28**	**13.62**	**7.21**	**6.55**
C18:2n6c	Linoleic	**74.19**	**70.09**	**51.41**	**32.59**	**69.95**	16.94
C20:0	Arachidic	n.d.	0.05	n.d.	n.d.	n.d.	n.d.
C20:1	Gondoic	2.11	0.24	n.d.	n.d.	n.d.	n.d.
C18:3n3	α-Linolenic	n.d	0.15	**6.78**	**2.24**	0.64	1.75
C20:2	Eicosadienoic	0.47	0.20	n.d.	n.d.	n.d.	n.d.
C22:0	Behenic	0.25	0.26	0.35	n.d.	0.35	n.d.
C20:3n6	Dihomo-gamma-linolenic	n.d.	n.d.	0.64	n.d.	n.d.	n.d.
C22:1n9	Erucic	0.08	0.15	0.10	n.d.	0.06	n.d.
C20:3n3	Eicosatrienoic	n.d.	n.d.	0.12	n.d.	n.d.	n.d.
C20:4n6	Arachidonic	0.08	0.09	n.d.	n.d.	0.09	n.d.
C23:0	Tricosylic	0.10	0.07	n.d.	n.d.	n.d.	n.d.
C22:2	Docosadienoic	1.32	1.75	n.d.	n.d.	n.d.	n.d.
C24:0	Lignoceric	0.73	0.50	0.09	n.d.	n.d.	n.d.
C20:5n3	Eicosapentaenoic	0.20	0.75	1.21	n.d.	1.21	37.31
C24:1	Nervonic	n.d.	n.d.	0.79	n.d.	n.d.	n.d.
C22:6n3	Docosahexaenoic	n.d.	0.08	n.d.	n.d.	n.d.	n.d.
SFA		16.29	14.92	17.80	35.29	20.48	37.44
MUFA		7.46	11.97	22.03	29.88	7.63	6.55
PUFA		**76.25**	**73.11**	**60.16**	34.83	**71.89**	**56.01**
UFA		**83.71**	**85.08**	**82.20**	**64.71**	**79.52**	**62.56**

UFA—unsaturated fatty acid; MUFA—mono-unsaturated fatty acids; PUFA—poly-unsaturated fatty acids, SFA—saturated fatty acids, n.d.—not detected. Bold values highlight the importance of the relevant contents.
